# A Study of the Differential Effects of Eicosapentaenoic Acid (EPA) and Docosahexaenoic Acid (DHA) on Gene Expression Profiles of Stimulated Thp-1 Macrophages

**DOI:** 10.3390/nu9050424

**Published:** 2017-04-25

**Authors:** Bénédicte Allam-Ndoul, Frédéric Guénard, Olivier Barbier, Marie-Claude Vohl

**Affiliations:** 1 Institute of Nutrition and Functional Foods (INAF), Pavillon des Services, 2440 Hochelaga Blvd, Laval University, Québec City, QC G1V 0A6, Canada; allamndoulbenedicte@gmail.com (B.A.-N.); Frederic.Guenard@fsaa.ulaval.ca (F.G.); 2 Laboratory of Molecular Pharmacology, CHU de Québec Research Center, Québec City, QC G1V 0A6, Canada; Olivier.barbier@crchul.ulaval.ca

**Keywords:** omega-3 fatty acids, inflammation, mRNA, microarrays, gene expression

## Abstract

**Background:** An appropriate intake of omega-3 (*n*-3) fatty acids (FAs) such as eicosapentaenoic and docosahexaenoic acid (EPA/DHA) from marine sources is known to have anti-inflammatory effects. However, molecular mechanisms underlying their beneficial effects on health are not fully understood. The aim of the present study was to characterize gene expression profiles of THP-1 macrophages, incubated in either EPA or DHA and stimulated with lipopolysaccharide (LPS), a pro-inflammatory agent. **Methods:** THP-1 macrophages were incubated into 10, 50 and 75 µM of EPA or DHA for 24 h, and 100 nM of LPS was added to the culture media for 18 h. Total mRNA was extracted and gene expression examined by microarray analysis using Illumina Human HT-12 expression beadchips (Illumina). **Results:** Pathway analysis revealed that EPA and DHA regulate genes involved in cell cycle regulation, apoptosis, immune response and inflammation, oxidative stress and cancer pathways in a differential and dose-dependent manner. **Conclusions:** EPA and DHA appear to exert differential effects on gene expression in THP-1 macrophages. Specific effects of *n*-3 FAs on gene expression levels are also dose-dependent.

## 1. Introduction

Inflammation is one of the responses of the immune system to harmful stimuli, such as pathogens or tumorous cells. The main purpose of inflammation is to eradicate the initial cause of cell injury, to clear necrotic cells and damaged tissue, and to initiate tissue repair. Although inflammation is a beneficial physiological process, it must be stopped after tissue repair [1]. However, a loss of control of this phenomenon can occur, contributing to the development of chronic inflammatory conditions such as rheumatoid arthritis, cardiovascular diseases (CVDs) or Alzheimer’s disease [2,3].

Scientific evidence supports the beneficial effects of marine omega-3 (*n*-3) fatty acids (FAs) such as eicosapentaenoic acid (EPA) and docosahexaenoic acid (DHA) on inflammatory disorders [4,5,6]. EPA and DHA have proven anti-inflammatory effects on a variety of immune cells [1,4]. Several studies have reported that *n*-3 FA anti-inflammatory effects are exerted by altering properties of immune cells [1,7,8,9,10]. More specifically, studies using macrophages and T cells have shown that *n*-3 FAs inhibit inflammatory cytokine production by modulating multiple transcription factors [11,12]. Recent studies have also shown that the incorporation of *n*-3 FAs into membrane phospholipids resulted in changes of gene expression profiles [8,9,12,13,14]. In addition, a whole-genome analysis demonstrated that a 26-week supplementation of healthy, elderly subjects with 1.8 g/day of EPA + DHA regulates hundreds of inflammatory genes in human immune cells [13]. The effect of a 6-week supplementation of 3 g/day *n*-3 FAs on gene expression profiles of peripheral blood mononuclear cells (PBMCs) from healthy subjects has been investigated. Pathway analysis revealed changes in expression levels of genes involved in inflammation and oxidative stress [15].

However, there are very few studies comparing specific effects of EPA and DHA on the gene expression profiles of immune cells. The aim of this study was to investigate the respective effect of EPA and DHA on the gene expression profile of lipopolysaccharide (LPS)-stimulated THP-1 macrophages.

## 2. Materials and Methods 

### 2.1. Reagents and Cell Lines

EPA, DHA and reagents for reverse transcription were obtained from Applied Biosystems (Oakville, ON, Canada). Cell culture media, Roswell Park Memorial Institute (RPMI) medium 1640, fetal bovine serum (FBS), phorbol 12-myristate 13-acetate (PMA), and dimethyl sulfoxide (DMSO) were purchased from Thermo Scientific (Walthman, MA, USA). Phosphate-buffered saline (PBS) solution was obtained from Life Technologies (Burlington, ON, Canada). Lipopolysaccharide (LPS) from *Escherichia coli* 0111:B4 (reference L2630) was purchased from Sigma (St. Louis, MO, USA).

### 2.2. Cell Culture and Fatty Acid Treatment

The human THP-1 cell line, an acute monocytic leukemia cell line (American Type Culture Collection (ATCC), Rockville, MD, USA), was cultured in RPMI 1640 media supplemented with penicillin (100 U/mL), streptomycin (100 µM/mL), and 10% FBS at 37 °C in a 5% CO_2_ incubator. Differentiation of monocytes into macrophages was induced with PMA. A total of 9 × 10^5^ cells per mL were seeded into 6-well plates, with 200 nM of PMA for 72 h. Then, non-attached cells were removed by aspiration and adherent cells were washed three times with PBS. Cells were then incubated with *n*-3 FAs for 24 h. After the 24-h incubation, 100 nM of LPS was added to the media for 18 h.

### 2.3. n-3 FAs and LPS Preparation

Prior to treatment, LPS was dissolved in PBS and diluted to a final concentration of 100 ng/mL. In preliminary studies, the effects of 10, 25, 50, 75 and 100 µM of EPA and DHA were tested. Low concentrations of 10 and 25 µM of *n*-3 FAs had comparable anti-inflammatory effects, whereas the highest concentration of 100 µM was toxic for the cells (data not shown). Thus, experiments were performed with concentrations of 10, 50 and 75 µM. Stock solutions of FAs (EPA–DMSO 33 × 10^4^ µM and DHA–DMSO 76 × 10^4^ µM) prepared in serum-free RPMI-1640 were diluted in culture media to obtain concentrations of 10, 50 and 75 µM. Fresh FAs and LPS were prepared for each experiment from frozen stock solutions. Cells were thereafter incubated with LPS and EPA or DHA for 24 h. Control conditions were THP-1 macrophages incubated with vehicle (DMSO) and LPS (LPS + macrophages). All treatments were performed in triplicate and the entire experiment was replicated independently three times.

### 2.4. RNA Isolation and Quantitative Real-Time Pcr

After 24 h, total RNA was extracted using RNeasy Mini Kit (Qiagen, Hilden, Germany), following manufacturer’s instructions. For quantitative real-time PCR analysis, RNA quality and integrity were tested on 1.5% agarose gel electrophoresis with ethidium bromide staining. Absorption spectroscopy at 260/280 was used to determine RNA concentration. cDNA was produced from RNA using the High Capacity Transcription Kit (Applied Biosystems). To validate results obtained with microarray analysis, expression levels of two inflammatory genes, *Tumor Necrosis Factor (TNFA)* and *Interleukin 6 (IL6)*, were measured using real-time PCR. PCR samples were normalized against 18S gene expression.

### 2.5. Transcriptomic Profiling

Total RNA (250 ng) was collected from each sample. cRNA quality was assessed using capillarity electrophoreses on an Agilent 2100 bioanalyzer. Expression levels of 51,281 mRNA transcripts, to investigate 47,323 genes, were assessed by Illumina Human HT-12 expression beadchips (Illumina, San Diego, CA, USA). Hybridization was carried out according to the manufacturer’s instructions at the McGill University and Génome Québec Innovation Centre (Montreal, QC, Canada).

### 2.6. Analysis of Microarray Data 

FlexArray software was used to analyse microarray data. Expression levels were normalized using Lumi and the robust multiarray average algorithm [16]. Quintile normalization and log2 transformation were performed. A significance analysis of microarrays (SAM) algorithm was used to assess differentially expressed transcripts between experimental conditions. The SAM algorithm consists of an adaptation of a *t* test for microarray data. In general, it assigns a score to a gene based on changes in gene expression relative to standard deviation of repeated measurements. Permutations of the repeated measurements are then used to estimate the false discovery rate [16]. A cut-off of *p* ≤ 0.05 was used to identify significantly regulated genes.

### 2.7. Network Identification and Canonical Pathway Analysis

Genes significantly regulated by EPA and DHA were analyzed using the Ingenuity Pathway Analysis (IPA) software (Ingenuity Systems, Redwood City, CA, USA). IPA classifies genes in lists based on their biological functions, and discloses networks connecting specific group of genes, allowing the linking of expression data to clinical endpoints to generate mechanistic hypotheses and to identify putative mechanisms. The significance value associated with functional analysis for a data set is a measure of the likelihood that the association between a set of functional molecules in the experiment and a given process or pathway is due to random chance. The *p* value is calculated using the right-tailed Fisher’s exact test with a cut-off of *p* ≤ 0.05. Using Cluster (cluster 3.0), we applied average-linkage hierarchical clustering to both differentially expressed genes and *n*-3 FA concentrations. Clustering results were analyzed and figures generated using Treeview (Treeview 1.1.6r4).

## 3. Results

### 3.1. Differentially Expressed Genes for EPA and DHA 

Several genes were found to be differentially expressed in THP-1 macrophages incubated in the presence of EPA or DHA versus the control condition (LPS+ macrophages) (Table 1). Briefly, a concomitant increase in the number of differentially expressed genes and in concentrations of *n*-3 FAs was seen. The number of differentially expressed genes was higher in macrophages incubated with 75, 50 and 10 µM of EPA, compared to corresponding DHA concentrations. 

### 3.2. Proportion of Modulated Pathways by EPA and DHA on the Proportion of Modulated Pathways Belonging to Different Families (Immune Response, Cell Cycle, Oxidative Stress and Other Pathways)

To determine the role of genes differentially expressed after incubation with EPA and DHA, a pathway analysis was performed. IPA was used to visualize gene expression data in the context of biological pathways. Pathways were regrouped into different families: ***immune response***, ***apoptosis***, ***cell cycle***, ***cancer***, ***oxidativestress*** and ***other pathways***. 

**Figure 1:**
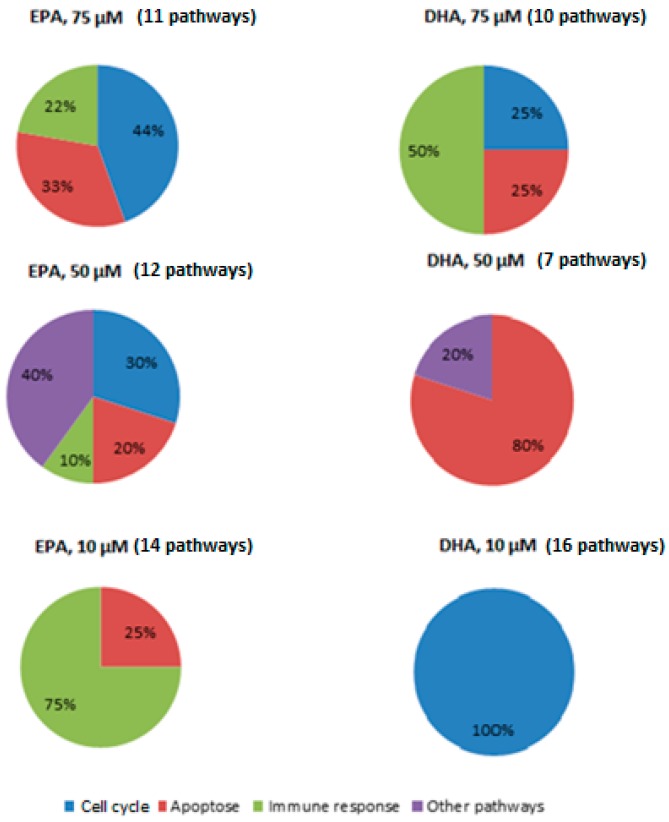
Proportion of activated pathways in LPS-stimulated macrophages incubated with EPA or DHA. The effect of 75, 50, and 10 µM of EPA or DHA on the proportion of modulated pathways belonging to different families (*immune response*, *apoptosis*, *cell cycle*, and *other pathways*) was investigated in LPS-stimulated macrophages.

Figure 1 shows the percentage of modulated pathways for each *n*-3 FA (EPA or DHA) and *n*-3 FA concentration (75, 50 or 10 µM), as compared to LPS + macrophages. First, the addition of 75 µM *n*-3 FAs modulated pathways involved in the *cell cycle* (EPA: 44%; DHA: 25%), *apoptosis* (EPA: 33%; DHA: 25%) and *immune response* (EPA: 22%; DHA: 50%). 

Second, the dose of 50 µM modulated pathways involved in *apoptosis* (EPA: 20%; DHA: 80%) and *other pathways* (EPA: 40%; DHA: 20%). Only EPA modulated pathways involved in *cell cycle* (30%) and *immune response* (10%) at 50 µM. 

Finally, while 10 µM of DHA modulated pathways involved in the *cell cycle* (100%), the same dose of EPA modulated pathways involved in *immune response* (75%) and *apoptosis* (25%). 

### 3.3. Impact of n-3 Fa Dose Reduction on Family Pathways

**Figure 2:**
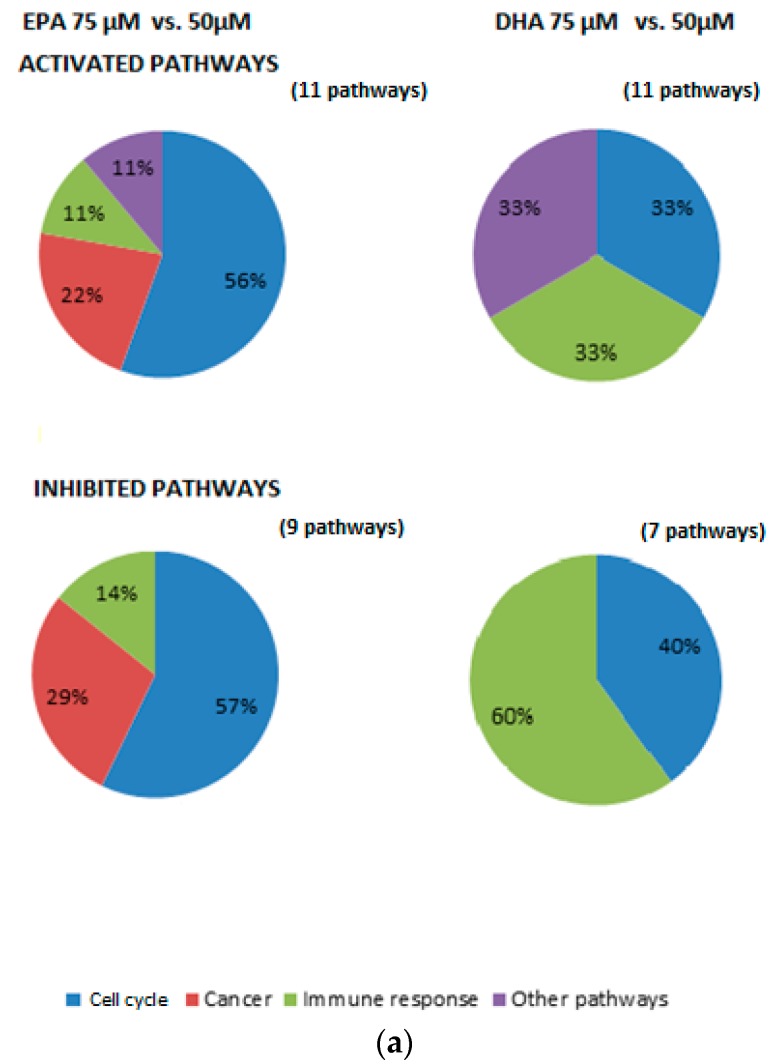

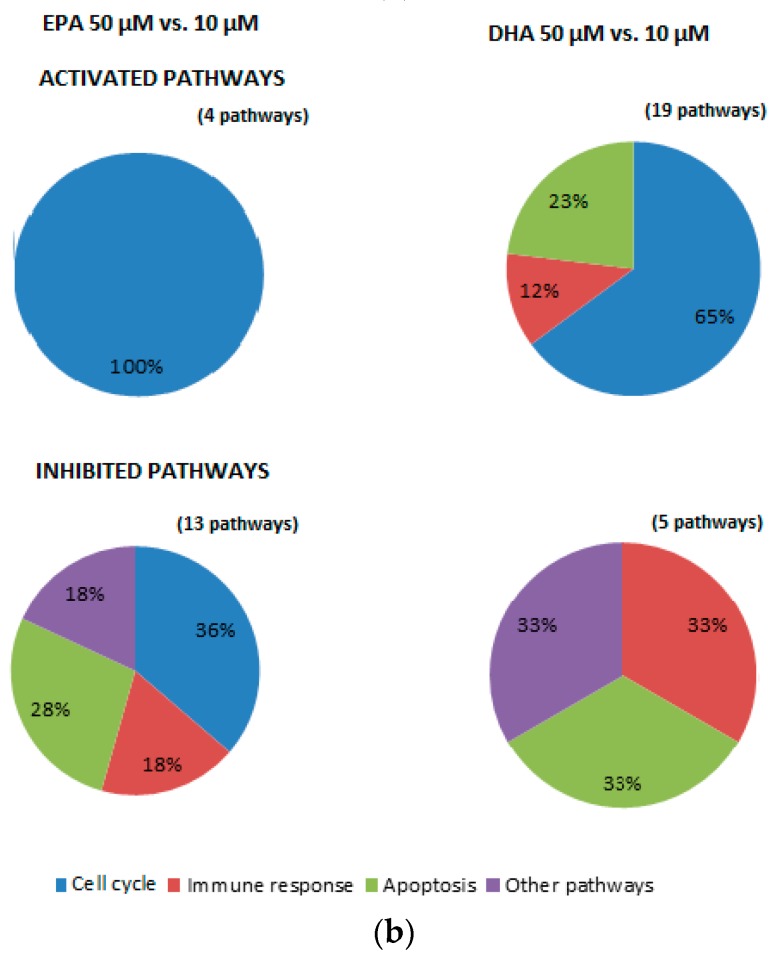

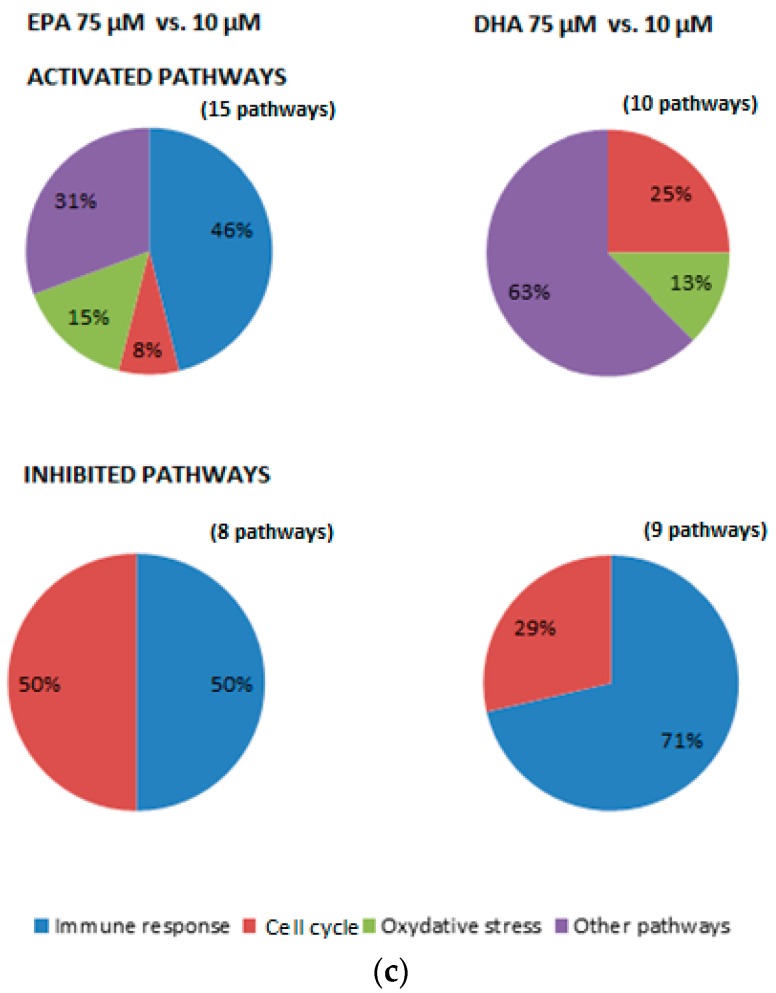
Effect of EPA and DHA dose reduction (75 µM versus 50 µM, 50 µM versus 10 µM and 75 µM versus 10 µM) on the proportion of modulated pathways belonging to different families (*immune response*, *cell cycle*, *oxidative stress* and *other pathways*) ((**a**–**c**), respectively).

The impact of *n*-3 FA dose reduction on the modulation of pathways was also investigated (Figure 2). First, an *n*-3 FA dose reduction from 75 to 50 µM induced the activation of pathways involved in the *cell cycle* (EPA: 56%; DHA: 33%), *immune response* (EPA: 11%; DHA 33%) and *other pathways* (EPA: 11%; DHA: 33%) (Figure 2a). This dose reduction also induced the inhibition of pathways involved in *cell cycle* (EPA: 57%; DHA: 40%) and *immune response* (EPA: 14%; DHA: 60%). Pathways involved in *cancer* were also activated (22%) and inhibited (29%) by a dose reduction of EPA (Figure 2a).

Second, an *n*-3 FA dose reduction from 50 to 10 µM mainly induced the activation of pathways involved in *immune response* (EPA: 100%; DHA: 65%) (Figure 2b). A dose reduction of DHA further activated pathways involved in *apoptosis* (23%) and *immune response* (12%). On the other hand, an inhibition of pathways related to *immune response* (EPA: 18%; DHA: 33%), *apoptosis* (EPA: 28%; DHA: 33%) and *other pathways* (EPA: 18%; DHA: 33%) was also observed. A dose reduction of EPA also induced the inhibition of pathways involved in *cell cycle* (36%).

Finally, incubating cells with 10 µM of *n*-3 FAs instead of 75 µM induced the activation of pathways involved in the *cell cycle* (EPA: 8%; DHA: 25%), *oxidative stress* (EPA: 15%; DHA: 13%) and *other pathways* (EPA: 31%; DHA: 63%) (Figure 2c). A dose reduction of EPA also activated pathways involved in *immune response* (46%). Meanwhile, this dose reduction also induced the inhibition of pathways involved in *immune response* (EPA: 50%; DHA: 71%) and the *cell cycle* (EPA: 50%; DHA: 29%).

### 3.4. Gene Expression Changes in Specific Pathways

To get a better understanding of EPA and DHA effects on the gene expression profile of THP-1 macrophages, differentially expressed genes were organized in specific pathways rather than in pathway families.

**Figure 3:**
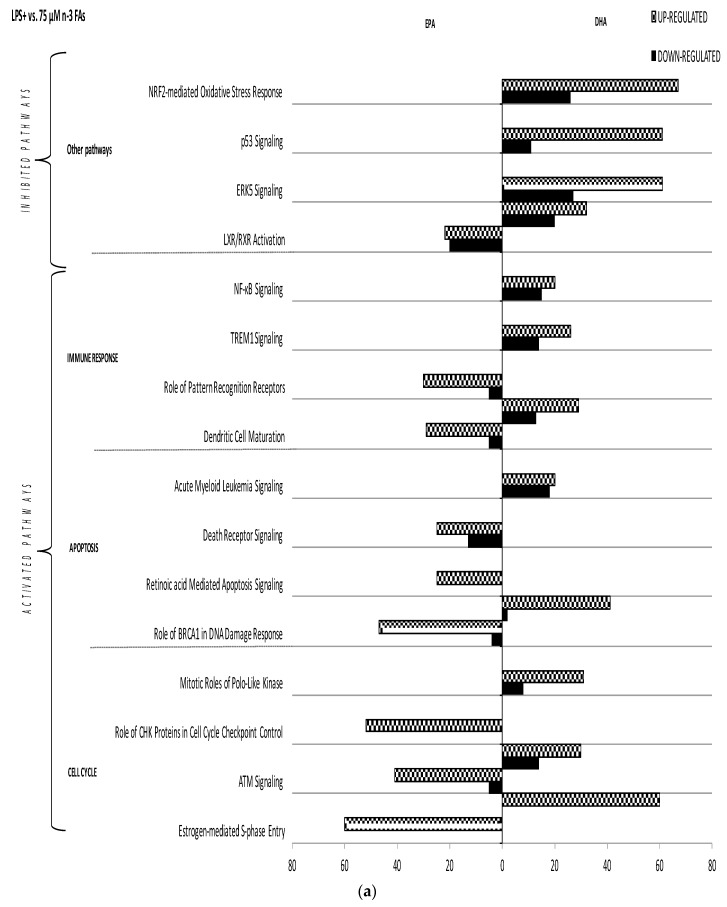

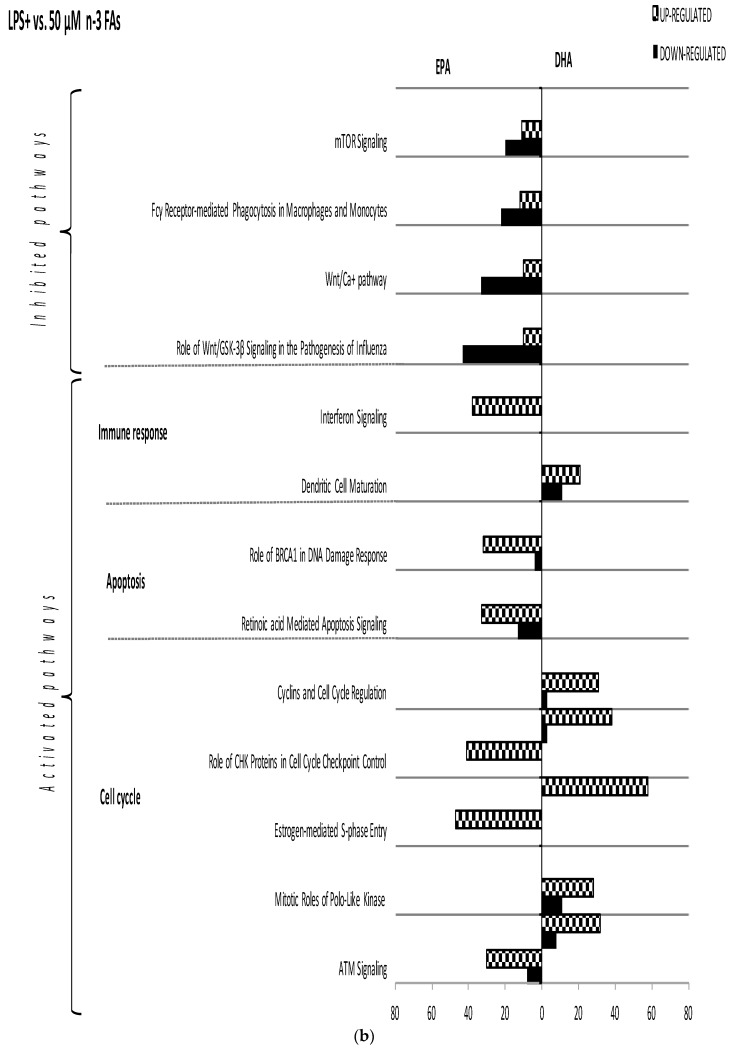

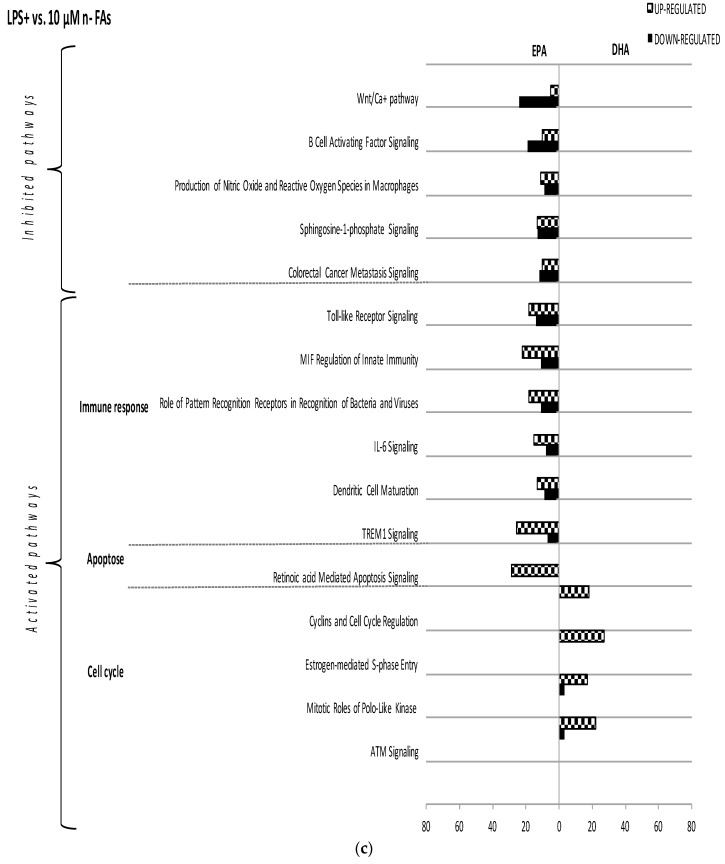
Impact of EPA and DHA on canonical pathways in LPS-stimulated macrophages. The effect of 75, 50 and 10 µ of EPA or DHA on gene regulation in LPS-stimulated macrophages is shown ((**a**–**c**), respectively). Differentially expressed genes are organized in canonical pathways regrouped into different families (*immune response*, *apoptosis*, *cellular cycle*, and *other pathways*). The amount of up- and down-regulated genes within each pathway is also shown (horizontal grey and black bars, respectively). MIF: macrophage migration inhibitory factor, IL: interleukin, TREM: Triggering receptor expressed on myeloid cells 1, ATM: Ataxia telangiectasia Mutated, NFKB: nuclear factor-kappa B, NRF2: Nuclear factor 2, ERK5: Extracellular-signal-regulated kinase 5, RXR: Retinoid X receptor, LXR: Liver X receptor.

First, LPS+ macrophages were compared to macrophages stimulated with LPS and incubated with 10, 50 or 75 µM of *n*-3 FAs. EPA and DHA regulated the expression of several specific pathways in a *n*-3 FA-specific manner (Figure 3). For instance, within the *cell cycle* family, 50 µM of EPA regulated genes involved in *ATM signaling*, *estrogen-mediated-S-phase entry* and *role of CHK proteins in cell cycle checkpoint control* pathways, whereas 50µM of DHA regulated genes involved in *ATM signaling*, *estrogen-mediated-S-phase entry*, *mitotic role of polo-like kinase* and *cyclins and cell cycle regulation* pathways.

Irrespective of the FA, the incubation of LPS+ macrophages with different *n*-3 FAs concentrations also had distinct effects on the modulated pathways (Figure 3). Nevertheless, some pathways were consistently modulated across the different concentrations. In the *cell cycle* family, EPA modulated *ATM signaling*, estrogen-mediated-S-phase entry and *role of CHK proteins in cell cycle checkpoint control* pathways at both 75 and 50 µM. Likewise, DHA modulated *ATM signaling*, *mitotic roles of polo-like kinase* and *estrogen-mediated-S-phase entry* pathways at 10, 50 and 75 µM. Moreover, the *cyclins and cell cycle regulation* pathway was also regulated by DHA at 50 and 10 µM. On the other hand, EPA modulated *role of pattern recognition receptors* and *dendritic cell maturation* pathways at 75 and 10 µM in the *immune response* family, whereas DHA activated the expression of genes involved in *dendritic cell maturation* at 75 and 50 µM. Finally, EPA regulated the *role of BRCA1 in DNA damage response* pathway at 75 and 50 µM, and the *retinoic acid-mediated apoptosis signaling* pathway at 10, 50 and 75 µM in the *apoptosis* family (Figure 3).

### 3.5. Impact of n-3 FA Dose Reduction on Specific Pathways

**Figure 4:**
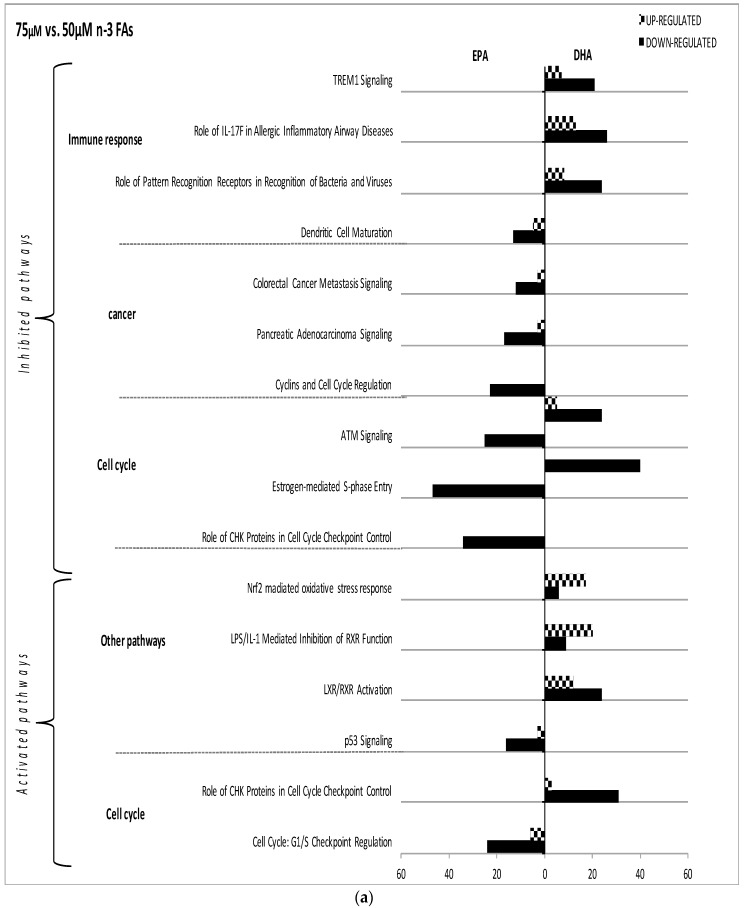

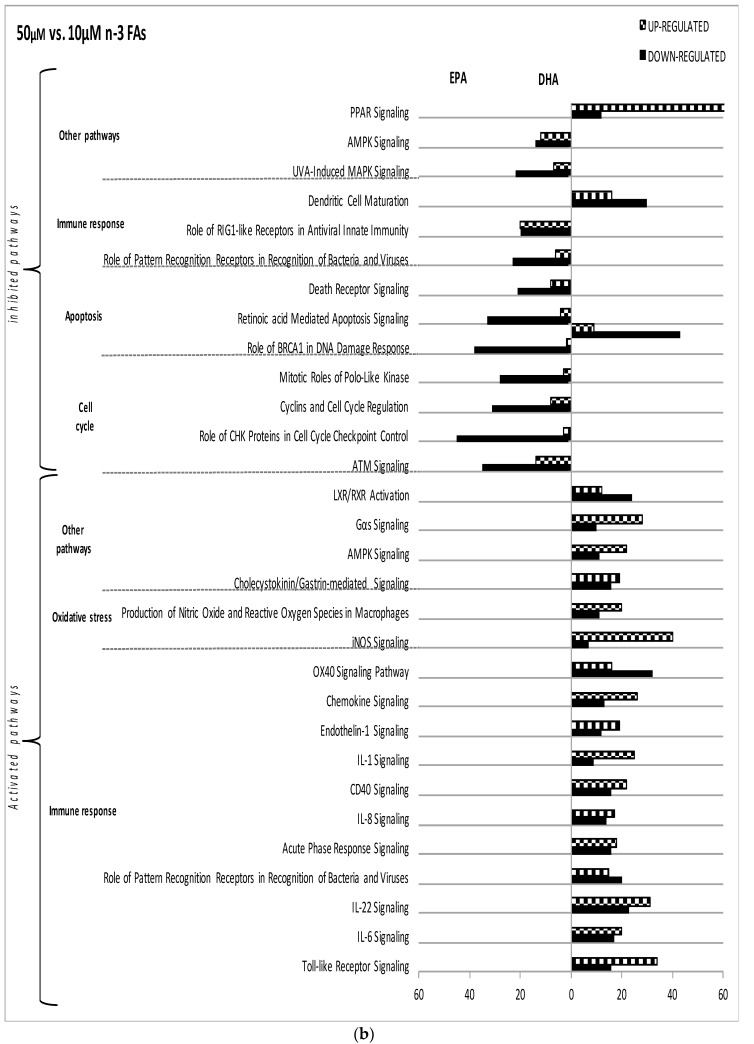

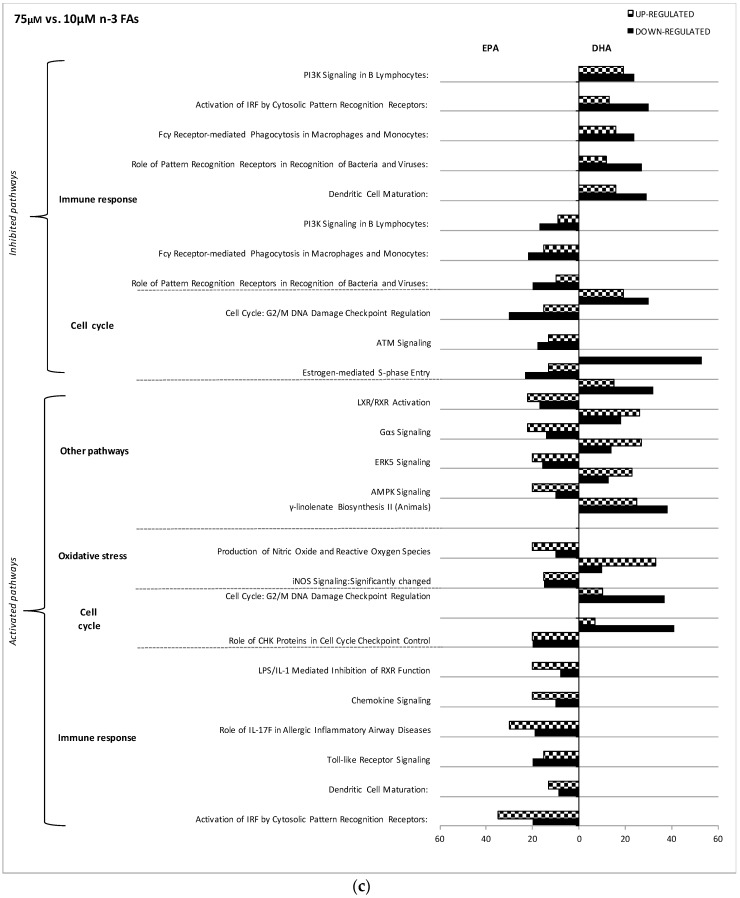
Impact of EPA and DHA dose reduction on canonical pathways in LPS-stimulated macrophages. The effect of EPA and DHA dose reduction (75 µM versus 50 µM, 75 µM versus 50 µM and 75 µM versus 10 µM) on gene regulation in LPS-stimulated macrophages is shown ((**a**–**c**), respectively). Differentially expressed genes are organized in canonical pathways regrouped into different families (*immune response*, *apoptosis*, *cellular cycle*, *oxidative stress* and *other pathways*). The amount of up- and down-regulated genes within each pathway is also shown (horizontal grey and black bars, respectively). PPAR: Peroxisome Proliferator-Activated Receptor, AMPK: AMP-Activated Protein Kinase, RIG1: RIG-I-like Receptor, INOS: Inductible Nitric Oxide Synthase, CD40: Cluster of Differentiation 40, IRF: Interferon Regulatory Factor, PI3K: Phosphoinositide 3-kinase, Fc gamma: Fc-Gamma Receptor, CHK: Checkpoint kinase.

Dose reduction of EPA and DHA regulated gene expression in a *n*-3 FA-specific manner (Figure 4). For example, in the *immune response* family, a dose reduction from 75 to 50 µM EPA modulated the *dendritic cell maturation* pathway, while the same dose reduction of DHA modulated the *TREM1 signaling* and *role of pattern recognition receptors* pathways.

Some pathways were modulated by different concentrations of the same *n*-3 FA. Dose reductions from 75 to 50 µM EPA induced the inhibition of *ATM signaling*, *estrogen-mediated-S-phase entry* and *cyclins and cell cycle regulation* pathways. DHA also inhibited *ATM signaling* and *estrogen-mediated-S-phase entry* at both dose reductions within the *cell cycle* family (Figure 4). 

### 3.6. Changes in the Expression of Genes Involved in Inflammation and Cell Cycle

**Figure 5:**
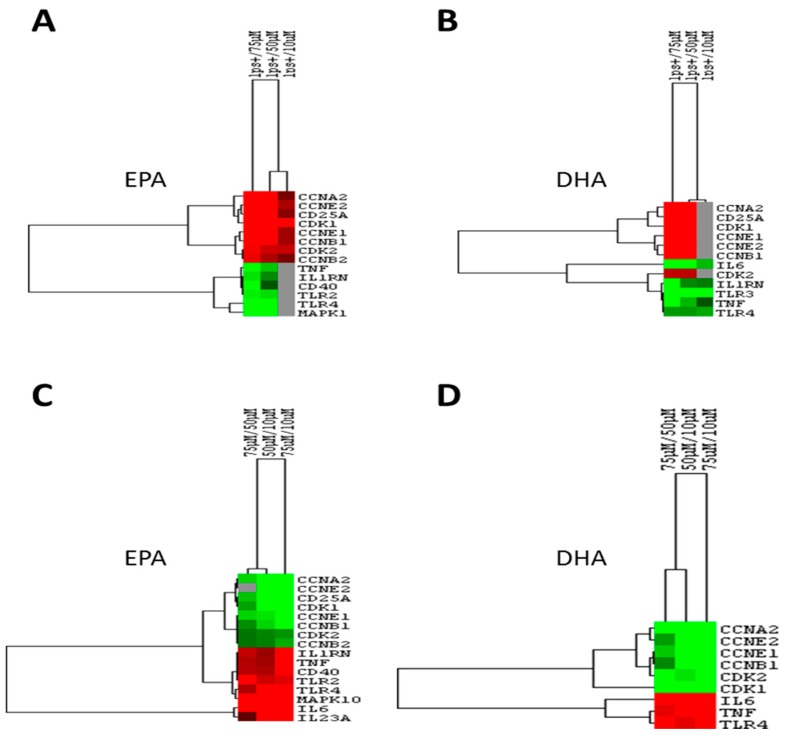
Panels (**a**) and (**b**) represent gene expression differences in LPS-stimulated macrophages (LPS+) versus LPS+ macrophages plus 10, 50 and 75 µM EPA or DHA. Panels (**c**) and (**d**) show comparisons in gene expression levels for different omega-3 (*n*-3) fatty acid (FA) concentrations. CCN: cyclin, CDK: cyclin-dependent kinase 2.

Since *immune response* and *cell cycle* were two of the most regulated family pathways, changes in expression levels of specific genes belonging to these families were analyzed (*immune response*: *CD40*, *IL1RN*, *TLR3*, *TLR4*, *CD40* and *TNFA*, and *cell cycle: CCNA2*, *CCNE2 CD25A*, *CCNB1*, *CCNB2*, *CCNE1*, *CDK2* and *CDK1*). A hierarchical analysis of microarray-based mRNA expression was computed and genes were clustered by similarity in gene expression magnitude (Figure 5).

Four major clusters arose from EPA analysis (Figure 5a). The first cluster regrouped *cell cycle* genes (*CCNA2*, *CCNE2 CD25A* and *CDK1*), as well as the second (*CCNB1*, *CCNE1*, *CDK2* and *CCNB2*). On the other hand, clusters three (*TNFA*, *CD40* and *IL1RN*) and four (*TLR4* and *CD40*) were composed of *immune response* genes.

The first (*CD25A* and *CCNA2*) and second (*CCNE2*, *CCNB1*) clusters arose from DHA analysis and assembled *cell cycle* genes (Figure 5b). The third cluster gathered *immune response* genes (*IL1RN*, *TLR3*, *TLR4*, and *TNFA*). Regarding the dose-effect, two major divisions arose among the three *n*-3 FAs concentrations tested (10, 50 and 75 µM) for both EPA and DHA (Figure 5a,b). The first cluster arose from the highest *n*-3 FA concentration (75 µM), and the second from the combination of 50 and 10 µM. It must be noted that 10 µM EPA did not influence the expression of *immune response* genes (Figure 5a), as compared to LPS alone. The same trend was observed with 10 µM of DHA but with *cell cycle* genes (Figure 5b). EPA appeared to be more potent to repress *cell cycle* genes than DHA at lower doses. The inverse phenomenon was observed for *immune response* genes. 

Clustering results for different concentrations of EPA (Figure 5c) and DHA (Figure 5d) showed that five and two major clusters arose among studied genes, respectively. Figure 5c shows five major clusters arising from EPA analysis. First (*CCNA2*, *CCNE2*, *CD25A*, and *CDK1*) and second (*CCNE1*, *CCNB1*, and *CCNB2*) clusters regrouped *cell cycle* genes. The last three clusters were composed of *immune response* genes (cluster 3: *IL1RN*, *CD40*, *TNFA*, *TLR2*; cluster 4: *TLR4* and *MAPK10*; cluster 5: *IL6* and *IL23A*). On the other hand, the two clusters arose from DHA analysis (Figure 5d) were composed of *cell cycle* (*CCNE2*, *CCNA2*, *CCNE1*, *CCNB1*) and *immune response* genes (*TNFA*, *TLR4*). *IL6* and *CDK1* were not included in any cluster. 

Regarding doses, two major clusters arose among the three *n*-3 FA concentrations. The first cluster included the 75/10 µM concentration, whereas the second one gathered the other two concentrations (75/50 µM and 50/10 µM). 

### 3.7. Validation of Microarray Analysis

**Figure 6:**
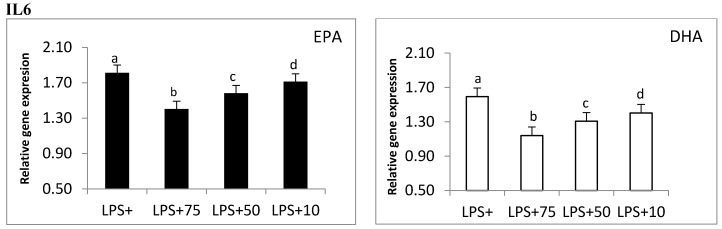

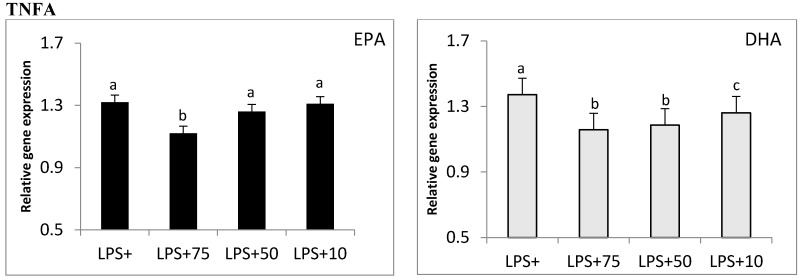
Changes in *TNFA* and *IL6* gene expression at different *n*-3 FAs concentrations were assessed using real-time PCR. PCR samples were normalized against 18S gene expression. Superscripts represent significant differences (*p* ≤ 0.05) between each concentration.

To confirm microarray analysis, changes in gene expression of *TNFA* and *IL6* with different *n*-3 FAs concentrations were assessed. A decrease on *IL6* and *TNFA* expression with increasing *n*-3 FAs concentrations was observed (Figure 6). In fact, a concentration of 75 µM inhibited gene expression in a more potent manner than 50 or 10 µM for both EPA and DHA. The concentration of 10 µM EPA did not have any effects on *TNFA* expression, whereas 10 µM of DHA significantly inhibited the expression of both genes (Figure 6). These results suggest that 10 µM of DHA was more efficient in reducing inflammatory genes than EPA, at least for the genes analyzed here.

## 4. Discussion

In this study, changes in gene expression profiles of THP-1 macrophages after incubation with LPS and different doses of EPA and DHA were investigated. To the best of our knowledge, this study is the first one comparing the specificity of EPA and DHA, as well as the dose-effect on gene expression profiles at a genome-wide level.

Several clinical studies reported the association between *n*-3 FA consumption and inflammatory disorders. Even though observational studies support the beneficial effect of *n*-3 FAs [17,18], results of intervention trials remain ambiguous, particularly among healthy subjects [19,20]. These inconsistencies may be partly explained by differences in study designs and patient selection criteria. EPA/DHA ratio and doses used are also of critical importance. Most intervention trials investigating the effect of *n*-3 FAs on inflammatory diseases used about 1 g/day of combined EPA + DHA [19]. To investigate the mechanism of action of EPA and DHA in a proper manner, it is important to understand that enough EPA and DHA must be provided. In this perspective, the optimal dose of EPA and DHA, as well as their optimal ratio in each pathological condition, must be extensively investigated to maximize their beneficial effects. Since EPA and DHA are thought to have different effects [10], it is important to study their action independently.

It is known that the beneficial effects of EPA and DHA on inflammation are partly mediated by the regulation of signalling pathways and gene expression in immune cells [21]. To obtain a comprehensive overview of the biological processes modulated by *n*-3 FAs, previous whole genome transcriptomic studies have been performed on PBMCs of human subjects receiving fish oil supplements (mixtures of EPA and DHA). These studies suggested a modulation of inflammatory, oxidative, endoplasmic reticulum stress and apoptosis regulatory pathways by *n*-3 FAs [13,22,23].

The aim of the present study was to go further by comparing the effects of different EPA and DHA concentrations on LPS-stimulated THP-1 macrophages using microarray analyses. IPA was used to cluster responsive genes according to main biological functions of their encoded protein. Both EPA and DHA markedly affected the expression of genes involved in cell cycle, immune response and inflammation, apoptosis, oxidative stress and cancer. Interestingly, although FA-specific effects were observed, several pathways were regulated by both EPA and DHA at 75 and 50 µM, suggesting that specific FA threshold concentrations are needed to regulate these pathways and influence gene expression. 

Although several pathways regulated by EPA and DHA were classified into the same family (*cell cycle*, *apoptosis* or *immune response*), specific pathways comprising each family were different for EPA and DHA. After a 6-week supplementation of 1.8 g/day EPA and DHA and the harvest of PBMCs on participants, Tsunoda et al. showed that, although several pathways regulated in each group were classified into the same larger family, all pathways were completely different between the two groups [24]. Herein, we show that even when the comparison of different *n*-3 FAs concentrations is done, the same families are regulated by the same EPA and DHA concentrations. However, pathways involved in each family differ for EPA and DHA. These results highlight that EPA and DHA are exerting their action through different molecular mechanisms.

*Immune response* and *cell cycle* were two of the most regulated family pathways. The effect of EPA and DHA on expression levels of several genes belonging to these families was then investigated. Genes involved in the cell cycle (*CCNA2*, *CCNE2*, *CD25A*, *CDK1*, *CCNE1*, *CCNB1*, and *CDK2*) were activated by EPA and DHA and a dose-effect was observed. Accordingly, the activation of these genes was more intense with higher doses. At lower concentrations, EPA appeared to have a greater impact than DHA on the regulation of these genes.

The comparison of different *n*-3 FAs doses revealed that a reduction of *n*-3 FAs concentrations triggered a down-regulation of genes involved in cell cycle. In a randomized controlled intervention trial with healthy subjects, Myhrstad et al. investigated the transcriptome profile of PBMCs after an intake of 1.6 g/day of EPA and DHA for 7 weeks. A significant modulation of genes related to apoptosis, cell cycle and endoplasmic reticulum stress was seen in patients receiving the *n*-3 FA supplementation. Among others, an increased expression of cyclins and cyclin-dependent kinases, e.g., *CCNE1* and *CDK2*, was observed. It is important to point out the fact that this study was performed among subjects who were not suffering from a chronic inflammation, thus demonstrating that even in non-inflammatory conditions, a mixture of EPA + DHA increases the expression of genes involved in cell cycle. In a study led by Verlagia et al. [10] T lymphocytes (TL) were incubated into 12.5 µM of EPA or DHA for 24 h. Microarray analysis showed that EPA and DHA markedly affected genes involved in cell cycle, defense and repair, apoptosis and DNA synthesis. Concretely, DHA up-regulated *CDC25A* and *CCNE*. The fact that this study was done on non-stimulated TL can explain the differences in results obtained. However, TL and macrophages belong to the immune cell family, and they are both involved in the inflammatory response. Interestingly, in this study, EPA and DHA also seemed to have a different action on non-stimulated LT, suggesting that a differential effect of these two FAs might be evident in non-inflammatory stage.

Regarding genes involved in inflammation, a dose-effect was observed. Decreasing *n*-3 FAs concentrations induced the expression of genes involved in immune response and inflammation. Several studies in healthy subjects, with varying *n*-3 FAs doses showed a reduction in cytokine production after ex vivo stimulation of inflammatory cells. For instance, in previous studies carried out by us and others [13,15], microarray analyses were performed on RNA extracted from PBMCs of subjects who received 1.8 g and 3 g EPA + DHA/day, respectively. The *n*-3 FA supplementation was associated with a decreased expression of inflammatory genes, including pro-inflammatory cytokines, oxidative stress and genes involved in the Nuclear factor-kappa B (NF-KB) pathway. Altogether, these data suggest that transcriptional regulation of genes involved in cell cycle progression and inflammation are modulated in a dose-dependent manner by *n*-3 FAs in THP-1 macrophages. The dose-effect was also shown in the clustering divisions based on *n*-3 FAs concentrations, strengthening the hypothesis that EPA and DHA exert their action in a dose-dependent manner. 

These FAs are also involved in the regulation of genes having a role in cancer. The in vitro inhibition of cell proliferation and cell growth in cancer by *n*-3 FAs has been previously demonstrated [25,26]. In human colon cancer cell lines, a target protein of cancer therapy and major cell regulators was found to be inhibited by DHA [26,27]. Differences found between *n*-3 FAs effects on carcinogenic and non-carcinogenic cells in vitro may be related to the fact that cancer cells are characterized by mutations in genes involved in cell death, cell cycle and repair functions, as well as because these cells have lost their normal function.

In conclusion, results of the present study strengthen the understanding of the effect of different doses of EPA and DHA on LPS-stimulated macrophages. The exposure of THP-1 macrophages to 75, 50 and 10 µM of either EPA or DHA induces changes in gene expression that may protect macrophages from an excessive inflammatory response. These changes are also FA-specific. Using an exploratory approach, biological pathways related to cell cycle, inflammation, apoptosis, cancer and oxidative stress were found to be enriched after incubation with EPA or DHA. These pathways are involved in normal cell functions and may ultimately influence whole body health. Most studies investigating the health benefits of *n*-3 FAs use a mixture of EPA and DHA rather than EPA and DHA separately. Results of the present study clearly suggest that EPA and DHA may exert their action through different mechanisms. It is thus crucial to investigate their combined and individual effects. It is also important to consider the dose of *n*-3 FAs used to obtain optimal benefits. Further studies in the field, involving transfection or proteomic techniques, are now required to better understand the specific effects of these *n*-3 FAs. In addition, intervention trials are also needed to elucidate the individual actions of *n*-3 FAs on chronic inflammatory disorders.

## Figures and Tables

**Table 1:** Differentially expressed genes in lipopolysaccharide (LPS)-stimulated macrophages (LPS+) incubated with eicosapentaenoic or docosahexaenoic acid (EPA/DHA).

Conditions	EPA	DHA
LPS+ versus 75 µM	4887	4875
LPS+ versus 50 µM	4674	4031
LPS+ versus 10 µM	2667	1577
